# Nonalcoholic Fatty Liver Disease Is Associated with Increased Atrial Fibrillation Risk in an Elderly Chinese Population: A Cross-Sectional Study

**DOI:** 10.1155/2018/5628749

**Published:** 2018-07-19

**Authors:** Yu Zhang, Peifei Li, Min Miao, Yi Liu, Yue Pan, Lu Xu, Zhongwei Zhu, Chengfu Xu, Lei Xu

**Affiliations:** ^1^Department of Gastroenterology, Ningbo First Hospital, No. 59 Liuting Street, Haishu District, Ningbo, Zhejiang Province, China; ^2^Ningbo University, College of Medicine, No. 818 Fenghua Road, Jiangbei District, Ningbo, Zhejiang Province, China; ^3^Department of Gastroenterology, The Affiliated Hospital of Ningbo University, School of Medicine, No. 247 Renmin Road, Jiangbei District, Ningbo, Zhejiang Province, China; ^4^Department of Gastroenterology, Zhenhai Lianhua Hospital, No. 168 Northern Tianyi Road, Jiaochuan Street, Zhenhai District, Ningbo, China; ^5^Department of Gastroenterology, The First Affiliated Hospital, College of Medicine, Zhejiang University, No. 79 Qingchun Road, Shangcheng District, Hangzhou, Zhejiang Province, China

## Abstract

**Background:**

Atrial fibrillation and nonalcoholic fatty liver disease are two pathological conditions that are highly prevalent worldwide and share multiple CVD risk factors. There are rare researches performed among elderly adults.

**Aims:**

We conducted this cross-sectional analysis of elderly adults (≥65 years) to investigate the association between atrial fibrillation and nonalcoholic fatty liver disease.

**Methods:**

We analyzed clinical data of the elderly adults (≥ 65 years) who had health examination in Zhenhai Lianhua Hospital, Ningbo, China, in 2014.

**Results:**

522 of the 1688 participants were diagnosed with nonalcoholic fatty liver disease, and 39 participants were diagnosed as having atrial fibrillation. Nonalcoholic fatty liver disease was associated with risk factors for AF in the elderly Chinese population (OR 1.95, 95% CI 1.03-3.69). Adjustments for age, gender, systolic blood pressure, fasting plasma glucose, *γ*-glutamyl transpeptidase, high-density lipoprotein, triglycerides, total cholesterol and albumin, nonalcoholic fatty liver disease, and prevalent atrial fibrillation remained statistically significant (OR 2.76, 95% CI 1.32-5.77).

**Conclusions:**

Our results show that nonalcoholic fatty liver disease is associated with an increased risk of atrial fibrillation in an elderly Chinese population.

## 1. Introduction

Atrial fibrillation (AF) is a growing public health problem [1]. Because of the aging population and improvements in cardiovascular treatments, its prevalence is expected to increase substantially over the next few decades [2]. AF has been reported to be associated with high rates of hospitalization and death [3]. Along with older age, there are many independent risk factors for AF like obesity, hypertension, diabetes, ischemic heart disease, heart failure, and valvular heart disease [4].

Nonalcoholic fatty liver disease (NAFLD) is one of the most prevalent liver diseases in the world whose prevalence ranges from 6% to 35%, with a median of 20% in the general population [5]. In recent years, an increasing body of evidence has indicated that NAFLD is linked to cardiovascular disease [6], myocardial abnormalities [7], left ventricular diastolic dysfunction [8], heart failure [9], aortic valve sclerosis, and so on [10].

NAFLD has also been observed to be significantly associated with AF in patients with type 2 diabetes [11, 12]. Furthermore, a cohort study showed that NAFLD was associated with an increased risk of prevalent AF in a middle-aged population [13]. However, whether the association between NAFLD and AF also holds true in the elderly population remains uncertain. Therefore, we conducted this cross-sectional study to explore the association between NAFLD and AF in an elderly Chinese population.

## 2. Materials and Methods

### 2.1. Participants

We conducted a cross-sectional study of the elderly adults (≥65 years old) who had undergone an annual physical examination at Zhenhai Lianhua Hospital, Ningbo, China, in 2014. 1688 participants (930 males and 758 females) with a median age of 72 (68-76) years were included in this analysis. This study excluded the following participants: (1) those with unknown alcohol intake or excessive alcohol intake; (2) those with unknown BMI or BMI≤18.0kg/m^2^; (3) those with incomplete basic physical data; (3) those with missing liver ultrasonic diagnosis; (4) those with unknown causes of chronic liver disease. This study was approved by the Hospital Ethics Committee. All the participants were verbally informed and agreed to participate in the study. Written informed consent was not required for the observational nature of the study.

### 2.2. Clinical Characteristics and Laboratory Data

Clinical examinations, including anthropometric and laboratory measurements, were performed using standard methods [14]. Height and weight were measured with basic clothing and without shoes. Body mass index (BMI) was calculated by the weight in kilograms divided by the square of height in meters. Blood pressure was recorded in a sitting position using a sphygmomanometer. Fasting blood samples were drawn from an antecubital vein for testing fasting plasma glucose (FPG), triglycerides (TG), total cholesterol (TC), high-density lipoprotein (HDL), low-density lipoprotein (LDL), and alanine aminotransferase (ALT), aspartate aminotransferase (AST), *γ*-glutamyl transpeptidase (GGT), serum uric acid, and albumin. All values were measured using an Olympus AU640 autoanalyzer (Olympus, Kobe, Japan) and standard methods.

### 2.3. Diagnosis of NAFLD

We diagnosed nonalcoholic fatty liver disease based on evidence of fatty liver according to abdominal ultrasonography using a Toshiba Nemio 20 sonography machine with a 3.5-MHz probe (Toshiba, Tokyo, Japan) and after excluding other etiology [15]. Routine ultrasonography evaluation of four intra-abdominal organs (liver, gallbladder, pancreas, and spleen) was performed by well-trained operators who were blind to the laboratory and clinical data. Diagnostic criteria of fatty liver are as follows: (i) increased liver brightness; (ii) diffuse hyperechogenicity of the liver compared to the kidneys; (iii) deep attenuation of hepatic echo; (iv) intrahepatic vessel borders and diaphragm [16].

### 2.4. Statistical Analysis

We made statistical analyses using SPSS 18.0 software for Windows (IBM SPSS, NY). Continuous variables were compared by Student's t-test or the Mann–Whitney* U* test and shown as the mean ±  SD (standard deviation) or the median and interquartile range. We compared categorical variables using the chi-square test or Fisher's exact test. We applied logistic regression analysis to assess the independent association between FLD and the prevalence of AF after adjustment for potential confounders. We considered a 2-tailed test and a* P* value less than 0.05 statistically significant.

### 2.5. Results

#### 2.5.1. Clinical Characteristics of the Included Patients

Of the 1688 elderly participants included in this study, 522 (30.9%, 269 males and 253 females) met the diagnostic criteria for NAFLD. Of the entire sample, 39 participants (2.3%, 30 males and 9 females) had persistent or permanent AF.

The clinical characteristics of patients with AF or without AF are shown in Table 1. Compared with the participants without AF, those with AF were older, were more likely to be male, and had significantly higher values of AST, GGT, and SUA. BMI, diastolic blood pressure (DBP), systolic blood pressure (SBP), ALT, TC, LDL, HDL, TG, albumin, and FPG did not significantly differ between the two groups. Notably, as also shown in Table 1, the participants with AF had a significantly greater prevalence of NAFLD than those without AF.

When the participants were stratified by NAFLD status (Table 2), we found that the group with NAFLD contained more males and was slightly younger than the group without NAFLD. The values for BMI, SBP, DBP, LDL, TG, AST, ALT, GGT, albumin, FPG, and SUA were higher among the participants with NAFLD; however, they had lower values of HDL. Importantly, as also shown in Table 2, there was a marked difference in the prevalence of AF among participants with or without NAFLD.

#### 2.5.2. Risk Factor Analysis for AF

As seen in Table 3, in the logistic regression analysis, relevant covariables were chosen as potential confounding factors based on their significance in the univariate analyses. In model 1, after adjustment for age and gender, the association between AF and NAFLD remained statistically significant (OR, 2.24; 95% CI 1.18–4.29). Further adjustment for SBP, FPG, GGT, HDL, and TG did not remarkably change the study group's status, as seen in model 2 (OR 2.72; 95% CI 1.30–5.67). In addition, age and GGT were independent predictors of AF in this model. In model 3, we added TC, and albumin based on model 3. In that model, the association between NAFLD and AF was still statistically significant (OR 2.76; 95% CI 1.32–5.77).

#### 2.5.3. Association between Liver Enzymes and the Prevalence Rate of AF

To explore the relationship between serum liver enzymes and the prevalence rate of AF, all the participants were classified into groups according to the upper limit of each serum liver enzyme concentration. For AST and ALT, we defined the normal level group as lower than 40U/L and elevated level group as higher than 40U/L. For GGT, the upper limit was 50U/L for males and 32U/L for females. The prevalence rate of AF in the groups with different levels of AST, ALT, and GGT was analyzed.

**Figure 1:**
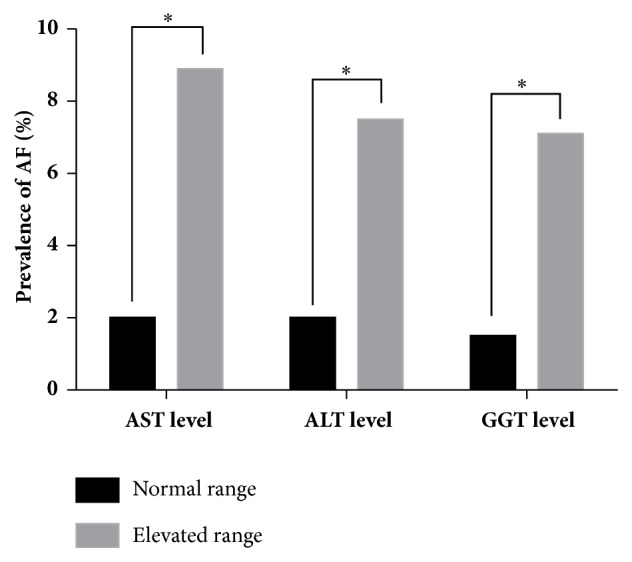
Prevalence of AF in an elderly Chinese population stratified by normal or elevated serum liver enzyme concentration. Normal range for AST or AST level: ≤40 U/L; elevated range for AST or ALT level: >40 U/L; normal range for GGT level in male: ≤50 U/L; elevated range for GGT in male: >50 U/L; normal range for GGT level in female: ≤32 U/L; elevated range for GGT in female: >32 U/L. *∗P* value <0.001/=0.004/<0.001 by the *χ*2 test in AST/ALT/GGT group.

**Figure 2:**
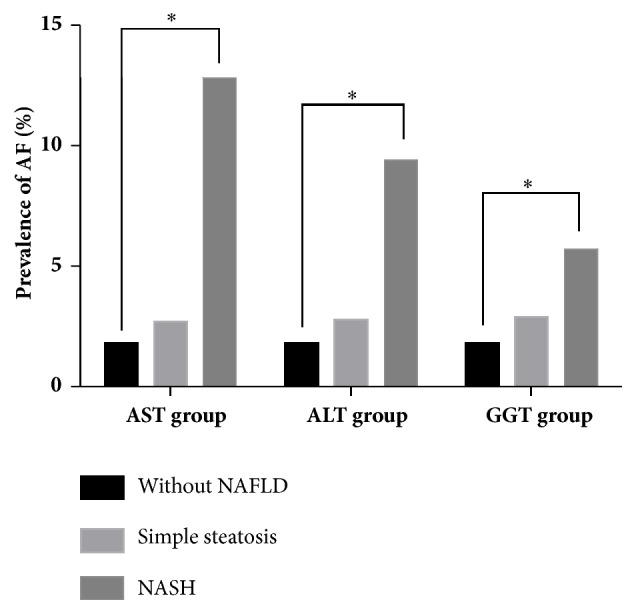
Prevalence of AF in an elderly Chinese population stratified by NAFLD status on ultrasound combined with normal or elevated serum AST/ALT/GGT concentration. Without NAFLD: without diagnosed NAFLD. Simple steatosis: NAFLD with normal range of AST/ALT/GGT. NASH: NAFLD with elevated range of AST/ALT/GGT. Normal range for AST or AST level: ≤40 U/L; elevated range for AST or ALT level: >40 U/L; normal range for GGT level in male: ≤50 U/L; elevated range for GGT in male: >50 U/L; normal range for GGT level in female: ≤32 U/L; elevated range for GGT in female: >32 U/L. *∗P* value =0.001/=0.003/=0.027 by the *χ*2 test in AST/ALT/GGT group.

As seen in Figure 1, there was an increasing trend of the prevalence rate of AF as serum liver enzyme increased. The prevalence rates of AF in normal and elevated serum AST groups are 2.0% and 9.6%. And this trend is significant (Figure 1;* P *<0.001). Meanwhile, the rates in normal and elevated serum ALT/GGT groups are 2.1% and 7.8%/1.6% and 7.2%, with significant result as well (Figure 2;* P* = 0.004/<0.001). These results showed that participants with higher serum liver enzyme are more likely to develop AF than those with lower one.

Additionally, we combined the serum liver enzyme and NAFLD status to further investigate the relationship between NAFLD and AF (Figure 2). The participants were classified into three groups: without NAFLD, NAFLD with normal concentration of AST/ALT/GGT, and NAFLD with elevated AST/ALT/GGT concentration. The classification boundary value was as shown in Figure 2. The prevalence rates of AF among the different groups were analyzed. Figure 2 shows that those with hepatic steatosis, irrespective of serum liver enzyme level, had the highest prevalence of AF. However, the presence of AF among those without hepatic steatosis on ultrasound was negligible.

## 3. Discussion

At present, NAFLD and AF are known to be two pathological conditions that are highly prevalent worldwide and share multiple CVD risk factors. In recent years, published studies about the association between AF and NAFLD (or liver transaminase concentrations) have increased [11–13, 17, 18]. The study by Targher et al. was limited to subjects with type 2 diabetes [11, 19], and the OPERA study focused on middle-aged participants [13]. What is more, after systematic analysis, several reviews claim similar conclusions and explain the mechanisms between two relative diseases [20–23]. As research performed among elderly adults is rare, we conducted this cross-sectional analysis of elderly adults (≥65 years) to investigate the association between NAFLD and AF.

The main finding of the present study was that NAFLD is associated with AF in an elderly Chinese population. The logistic regression also showed that NAFLD and prevalent AF were correlated (OR 1.95, 95% CI 1.03-3.69). Furthermore, the serum transaminase concentration was significantly associated with AF.

It is unknown whether these two diseases just share common pathophysiologic mechanisms or the association between them is causative. As NAFLD is a known risk factor for a wide range of cardiovascular diseases, it is reasonable to deduce that there may be a causal link. The following could be part of the explanation for our findings.

First, liver transaminases may link NAFLD and AF. The Framingham Heart Study demonstrated an independent relationship in the general adult population between liver transaminase concentrations and the risk of new-onset AF [24]. Targher et al.'s research proposed that, in subjects with type 2 diabetes, GGT was the only liver enzyme that was significantly associated with the prevalence of AF [12]. A similar result was found in an elderly Chinese population. GGT is a systemic marker of NAFLD [17]. The GGT level cannot be independent of NAFLD, so the development of the two diseases may be parallel. In addition, in our study, AST and ALT showed the same trend as GGT with the prevalence of AF. Furthermore, it has been proven that ALT is independently associated with an increased risk of cardiovascular related mortality, and ALT is the most specific marker of liver pathology [25–27]. However, Wang et al. supposed that the inflammatory response may be weaker in the aged liver [28] because lower ALT has been associated with increased mortality in the elderly [29]. Like Wang et al.'s study, the present investigation reported no mortality data. However, we must pay attention to high levels of ALT, especially in patients with NAFLD. As AST is produced not only in the liver, but also in the myocardium, it increases both NAFLD and AF in patients with both conditions. This is consistent with our results. Therefore, good control of liver transaminase concentrations may help to reduce the mortality of AF.

Second, NAFLD provokes systemic inflammation to aggravate AF. The accumulation of impaired lipoprotein and hepatic lipid and increased oxidative stress in hepatic cells may induce oxidative stress and cause the secretion of inflammatory factors [30, 31]. Previous studies have shown that chronic inflammation and oxidative stress are important risk factors for AF [32, 33]. Notably, many inflammatory factors produced by NAFLD [34] may cause AF. Furthermore, AF has been reported to be an trigger of an inflammatory environment [33], thus creating a vicious cycle. Remarkably, lipoprotein (HDL, LDL) can prevent the adhesion of bacteria and protest against endotoxemia or inflammation [35, 36]. The persistence of low levels of HDL and LDL could cause further lipoprotein consumption. These conclusions were consistent with our results shown in Table 1: HDL cholesterol, LDL cholesterol, and triglycerides were all lower in group with AF than in the group without AF.

Third, as NAFLD includes multiple cardiovascular risk factors, it may induce AF directly through several mechanisms. Some research has shown that NAFLD can cause left ventricular diastolic dysfunction [8, 37], and other studies have indicated that NAFLD alters atrial conduction properties [7, 38]. Fat may accumulate in not only the liver tissue, but also other viscera and organs. When it settles in the pericardium or myocardium, the diastolic function of the ventricle or atria is harmed [39, 40]. Moreover, increased fat can infiltrate the atrial septum and lead to electromechanical changes. It is important to provide health education to elderly patients with NAFLD to ensure that they develop healthier daily habits and get proper exercise.

Finally, many studies have reported that NAFLD is an independent risk factor for autonomic dysfunction [41–43]. Additionally, Sun et al. showed results that correspond to those of our study [43]. There are also other reports that showed that autonomic dysfunction is a risk factor for AF [44, 45]. These results may partly explain the relationship between NAFLD and AF.

Though there have been many articles showing similar information as shown in this study, the unique part is as follows: Firstly, research performed among elderly adults is rare and the elderly population is increasing worldwide [46]. Since China became a aging country in 2000, the development trend of the aging population, the health of the elderly population, and related social problems have all been taken seriously. It is our responsibility to pay more attention to the health of the elderly. Secondly, the prevalence of NAFLD among Chinese and foreigners is different [47, 48]. Since many international standards are not fully applicable to Chinese people, this study can provide health guidance for the larger Chinese population.

There are some limits to our study. First, though ultrasound-based diagnosis of NAFLD is widely used in clinic as a noninvasive and cost-effective method for hepatic steatosis screening, it cannot replace the gold standard, pathological study, for disease diagnosis. Second, with consideration of the prevalence of these diseases, the sample of our study is small. If we can get more support from the medical center, we will carry out multicenter or cohort research in the future to further confirm and improve our conclusions. At present, there are many formal researches for us to further validate the conclusions. Third, we diagnose AF according to resting electrocardiograms for absence of 24-hour dynamic electrocardiogram which is more precise but more difficult to accomplish during health examinations. Fourth, as our data was cross-sectional, we did not know the most recent mean levels of some laboratory results for each participant. As formal cohort studies have shown, NAFLD is associated with an increased risk of prevalent AF [12, 13, 49]. What is more, Zhou YQ et al. [20] and Ding YH et al. [21] illustrated possible pathological mechanisms responsible for the association between NAFLD and increased risk of AF. Therefore, we can draw a conclusion regarding cause and effect.

In conclusion, NAFLD is associated with AF in an elderly Chinese population. In addition, based on previous studies, we can conclude that NAFLD is associated with an increased AF risk in an elderly Chinese population.

## Figures and Tables

**Table 1:** Clinical characteristics of study participants stratified by AF status.

Characteristic	All (n=1688)	Without AF (n=1649)	With AF (n=39)	t value	*P-*value
Gender (n) (male/female)	930/758	900/749	30/9	7.689^a^	**0.006 **
Age (years)	72 (68-76)	72 (68-76)	76 (71-79)	3.091^b^	**0.002 **
Body mass index (kg/m^2^)	23.8±2.9	23.8±2.9	24.4±3.5	1.132	0.265
Systolic blood pressure (mmHg)	135.3±17.3	135.4±17.3	131.4±18.5	-1.422	0.155
Diastolic blood pressure (mmHg)	74.6±10.2	74.6±10.2	76.8±10.0	1.360	0.174
Total cholesterol (mmol/L)	4.9±1.0	4.9±1.0	4.7±1.3	-1.068	0.292
High-density lipoprotein (mmol/L)	1.7±0.4	1.7±0.4	1.7±0.4	0.176	0.860
Low-density lipoprotein (mmol/L)	2.6±0.8	2.6±0.8	2.4±1.0	-1.096	0.280
Triglycerides (mmol/L)	1.15 (0.85-1.62)	1.15 (0.86-1.62)	1.05 (0.72-1.56)	1.164^b^	0.224
Aspartate aminotransferase (U/L)	23 (20-27)	23 (20-27)	26 (21-36)	2.144^b^	**0.032 **
Alanine aminotransferase (U/L)	17 (13-23)	17 (13-23)	18 (14-27)	1.140^b^	0.254
*γ*-glutamyl transpeptidase (U/L)	21 (16-31)	21 (16-31)	35 (23-78)	4.752^b^	**0.001 **
Albumin (g/L)	45.5±2.7	45.5±2.7	45.2±2.3	-0.670	0.503
Fasting plasma glucose (mmol/L)	5.34 (4.93-5.96)	5.33 (4.93-5.96)	5.52 (4.85-5.94)	0.301^b^	0.763
Serum uric acid (*µ*mol/L)	340±85.9	339±85.2	394±100.0	3.932	**0.001 **
NAFLD (%)	30.9	30.6	46.2	4.335^a^	**0.037**

Data are expressed as mean ± SD, median (IOQ), or percentage; a indicates *χ*2 test; b indicates Mann–Whitney U test. NAFLD, nonalcoholic fatty liver disease; AF, atrial fibrillation.

**Table 2:** Clinical characteristics of study participants stratified by NAFLD status.

Characteristic	Without NAFLD (n=1166)	With NAFLD (n=522)	t value	*P*-value
Gender (n) (male/female)	661/505	269/253	3.876	**0.049 **
Age (years)	72 (68-77)	71 (68-75)	-3.091^b^	**0.002 **
Body mass index (kg/m^2^)	23.0±2.5	25.8±2.8	19.452	**0.001 **
Systolic blood pressure (mmHg)	134.2±17.4	137.7±16.9	3.853	**0.001 **
Diastolic blood pressure (mmHg)	74.0±10.1	76.1±10.3	4.046	**0.001 **
Total cholesterol (mmol/L)	4.89±0.99	4.96±1.00	1.314	0.189
High-density lipoprotein (mmol/L)	1.7±0.4	1.5±0.3	-11.008	**0.001 **
Low-density lipoprotein (mmol/L)	2.6±0.8	2.7±0.8	1.542	0.123
Triglycerides (mmol/L)	1.02 (0.78-1.45)	1.47 (1.10-2.04)	-13.114^b^	**0.001 **
Aspartate aminotransferase (U/L)	23 (20-27)	24 (20-30)	-2.936^b^	**0.003 **
Alanine aminotransferase (U/L)	16 (12-21)	20 (15-28)	-9.946^b^	**0.001 **
*γ*-glutamyl transpeptidase (U/L)	20 (16-29)	26 (19-37)	-9.656^b^	**0.001 **
Albumin (g/L)	45.3±2.7	45.8±2.7	3.400	**0.001 **
Fasting plasma glucose (mmol/L)	5.26 (4.86-5.82)	5.52 (5.12-6.27)	-6.957^b^	**0.001 **
Serum uric acid (umol/L)	329±81.6	367±89.2	8.722	**0.001 **
AF (%)	1.8	3.4	4.335a	**0.037**

Data are expressed as mean ± SD, median (IOQ), or percentage; a indicates *χ*2 test; b indicates Mann–Whitney U test. NAFLD, nonalcoholic fatty liver disease; AF, atrial fibrillation.

**Table 3:** Association between NAFLD and risk of prevalent AF in patients in an elderly population. Model 1: age and gender; model 2: age, gender, systolic blood pressure, fasting plasma glucose, *γ*-glutamyl transpeptidase, high-density lipoprotein, and triglycerides; model 3: model 2 plus total cholesterol and albumin.

NAFLD (yes compared with no)	OR (95% CI)	*P*-value
Unadjusted model	1.95 (1.03-3.69)	0.041
Adjusted model 1	2.24 (1.18-4.29)	0.014
Adjusted model 2	2.72 (1.30-5.67)	0.008
Adjusted model 3	2.76 (1.32-5.77)	0.007

Other independent predictors of AF in model 2		
Age	1.11 (1.05-1.18)	0.001
*γ*-glutamyl transpeptidase	1.01 (1.01-1.02)	0.001

NAFLD, nonalcoholic fatty liver disease; AF, atrial fibrillation.
